# A Comparison of Sugar Intake between Individuals with High and Low Trait Anxiety: Results from the NutriNet-Santé Study

**DOI:** 10.3390/nu13051526

**Published:** 2021-04-30

**Authors:** Junko Kose, Adrienne Cheung, Léopold K. Fezeu, Sandrine Péneau, Charlotte Debras, Mathilde Touvier, Serge Hercberg, Pilar Galan, Valentina A. Andreeva

**Affiliations:** 1 Nutritional Epidemiology Research Group (EREN), Sorbonne Paris Nord University, INSERM U1153/INRAE U1125/CNAM, Epidemiology and Statistics Research Centre (CRESS), University of Paris, 93017 Bobigny, France; j.kose@eren.smbh.univ-paris13.fr (J.K.); acheu084@uottawa.ca (A.C.); l.fezeu@eren.smbh.univ-paris13.fr (L.K.F.); s.peneau@eren.smbh.univ-paris13.fr (S.P.); c.debras@eren.smbh.univ-paris13.fr (C.D.); m.touvier@eren.smbh.univ-paris13.fr (M.T.); s.hercberg@eren.smbh.univ-paris13.fr (S.H.); p.galan@eren.smbh.univ-paris13.fr (P.G.); 2 Department of Psychiatry, The Ottawa Hospital, Ottawa, ON K1Y 4E9, Canada; 3 Department of Public Health, AP-HP Avicenne Hospital, 93017 Bobigny, France

**Keywords:** diet, dietary sugars, carbohydrates, anxiety, mental health, epidemiological study

## Abstract

(1) Background: Dietary carbohydrates are likely correlated with mental health in general, and with anxiety in particular. Our aim was to investigate the cross-sectional relationship between trait anxiety and carbohydrate (especially sugar) intake in a large sample derived from the general French population. (2) Methods: The analyses included 20,231 non-diabetic adults enrolled in the NutriNet-Santé e-cohort, who had completed the trait anxiety subscale of the Spielberger State-Trait Anxiety Inventory (T-STAI, 2013–2016) and who were subsequently divided into high and low trait anxiety groups (T-STAI cut-off of 40 points). Sugar-rich food and macronutrient intake was calculated from ≥3 self-administered 24-h dietary records. The association between trait anxiety and carbohydrate intake was evaluated by ANCOVA according to age category (<45 and ≥45 years). (3) Results: In the full sample, 7942 (39.3%) individuals fell into the high trait anxiety category. They were more likely to be women (82.2% versus 69.2%; *p* < 0.0001) and younger (mean age 51.6 versus 55.1 years; *p* < 0.0001) compared to the low trait anxiety group. In fully-adjusted models, high-anxiety individuals aged under 45 years had significantly higher mean consumption of added simple sugars (43.9 versus 42.3 g/d; *p* < 0.0007), whereas those aged over 45 years with high trait anxiety had significantly lower mean consumption of fruit (214.0 versus 219.5 g/d; *p* < 0.02) compared to their low-anxiety counterparts. (4) Conclusions: This cross-sectional study revealed modest age-specific associations between anxiety status and sugar intake among adults. Prospective studies with representative samples are needed to explore potential bi-directionality of the observed associations.

## 1. Introduction

Mental illnesses account for a substantial and growing proportion of the global disease burden. In particular, pooled estimates suggest that lifetime anxiety disorders affect nearly 17% of adults, with the prevalence surpassing 28% in some Western countries [1]. These disorders are among the top 10 causes of years lived with disability in all World Health Organization (WHO) regions; moreover, from 2007 to 2017, the number of years lived with disability attributable to anxiety disorders increased by 12.4% for females and by 13.6% for males [2]. These disorders are highly comorbid with other mental [3] and chronic physical conditions [4]. Therefore, it is crucial to address anxiety disorders by means of epidemiological and prevention research.

In that context, interest in the field of nutritional psychiatry investigating the association between nutritional factors and psychiatric conditions has been gaining momentum. With regards to anxiety, significant associations have already emerged with fruit/vegetable intake [5,6], and dietary patterns/dietary quality [7,8]. Dietary intake influences the gut microbiome composition, immune system function, oxidative stress levels, and inflammation, all of which are regarded as plausible mechanisms of the relationship [9]. Moreover, there is some evidence of a positive relationship of trait anxiety with fasting glucose and insulin levels in adults [10].

Recently, research into the link between dietary sugars and mental illness has started to grow. For instance, large cohort studies reported adverse effects of added sugars, sugar-sweetened beverage consumption or a high glycaemic index diet on depression [11,12,13], which displays a high level of comorbidity with anxiety disorders [14]. Moreover, there are studies suggesting that a high sugar intake is significantly associated with low-grade inflammation [15], which, in turn, might affect brain activity related to anxiety [16]. The consumption of high-sugar foods likely attenuates the psychological (i.e., anxiety, depressed mood) effects of stress via actions in the periphery (i.e., glucocorticoid receptor signaling in adipose tissue) and in the brain (i.e., plasticity in brain reward regions) [17]. In turn, some complex carbohydrates are fermented by gut microbes, thus, producing short-chain fatty acids that appear to have anti-inflammatory, protective effects [18]. 

Nevertheless, the number of epidemiological studies of the sugar intake–anxiety link is limited and the findings are inconsistent. Some cross-sectional studies reported significant positive associations of anxiety with the consumption of soft drinks in Chinese adolescents [19], with dietary patterns featuring increased intake of added sugars and saturated fatty acids in Greek older adults [20], and with the consumption of added sugars in U.S. college students [21]. Other studies, however, did not find significant associations between anxiety and various measures of sugar consumption [22]. Limitations of existing studies include a focus on a single food/beverage group, potential classification bias regarding dietary intake and/or anxiety status, small sample sizes, and use of specific, relatively homogeneous samples such as students or the elderly. Therefore, the aim of the present study was to assess the association between anxiety status and sugar intake in a large adult sample recruited from the general population, using validated data collection instruments.

## 2. Materials and Methods

### 2.1. Study Population

NutriNet-Santé is an ongoing, web-based prospective cohort study launched in 2009 in France. Its overarching aims are (1) to study the direct and indirect relationship between nutrition and physical/mental health, and (2) to investigate determinants of dietary habits and nutritional status. Information about the study design and methodology is published elsewhere [23]. Briefly, eligible adults aged 18 years and older with Internet access are recruited from the general population via multimedia campaigns. The NutriNet-Santé study was approved by the Institutional Review Board of the French Institute for Health and Medical Research (INSERM # 00000388FWA00005831) and by the National Commission on Informatics and Liberty (CNIL # 908450 and # 909216). NutriNet-Santé is registered with # NCT03335644 at ClinicalTrials.gov.

At inclusion, participants complete a set of five self-report questionnaires: diet (e.g., 24-h dietary records for three randomly selected days over two weeks; described below), physical and mental health status, anthropometrics, physical activity, and socio-demographic and lifestyle characteristics. 

### 2.2. Sugar Intake 

In NutriNet-Santé, dietary intake is assessed at inclusion and on a biannual basis thereafter, via 24-h dietary records, previously validated against dietitian interviews and various biomarkers of nutritional status [24,25]. In this analysis, dietary sugar intake was the outcome of interest. It was evaluated by means of ≥3 × 24-h dietary records for each participant. The dietary data came from records completed within a 2.5-year window around the date of completion of the anxiety questionnaire (described below). Participants were asked to report all consumed food, beverages, composite dishes, the portion size/quantity and the recipe and/or seasoning for each item, along with the meal setting (place, time, etc.). Portion sizes were estimated using validated photographs [26], standard serving containers or directly in g or mL. A French food composition table featuring >3500 different items was used to calculate mean daily energy and nutrient intake [27]. All dietary data were weighted in order to account for weekday versus weekend consumption practices. Dietary energy under-reporting was identified via Black’s method [28], taking into account the individual’s age, sex, weight, height, physical activity level, and basal metabolic rate. Those with energy under-reporting were excluded from the analysis in order to strengthen the validity of dietary data. In addition, given the objectives of the study, individuals with prevalent or incident diabetes type 1 or type 2 and/or pregnant women were ineligible for analysis.

Next, two sugar-rich food groups were defined for this study: (1) “sweet food/beverage group” including fresh and dried fruit, milk-based desserts, sweetened breakfast cereals and cereal bars, various sugar-rich food products (e.g., cakes, cookies, pastries, etc.), sweetened non-alcoholic beverages, and 100% fruit juice; and (2) “sweet food group except fresh fruit” including the same types of food/beverages as group 1 except for fresh fruit. 

Overall, the following outcome variables related to sugar intake were analysed: percentage energy from carbohydrates, mean total carbohydrates (g/d), mean complex carbohydrates (g/d), mean simple sugars (g/d), mean added simple sugars (g/d), mean sweet food/beverage intake (Group 1; g/d), mean sweet food/beverage intake except fresh fruit (Group 2; g/d), and mean fresh fruit intake (g/d). In the present study, added simple sugars are those that are added during food processing, whereas simple sugars include both added simple sugars and simple sugars naturally-present in food, such as fructose in fruit and lactose in milk.

### 2.3. Trait Anxiety

Trait anxiety was the main exposure in this analysis. It is a relatively stable personality disposition characterized by a tendency to exhibit tension and apprehension (i.e., state anxiety) across a wide range of situations [29]. It was evaluated by means of self-reports on the trait anxiety subscale of the State-Trait Anxiety Inventory Form Y (T-STAI). That questionnaire was administered between 2013 and 2016 as part of the NutriNet-Santé follow-up, with each participant completing it only once. STAI is one of the most widely used tools for evaluating general anxiety proneness (as a state and as a trait), distinguishing it from depression [29]. State and trait anxiety are assessed via separate sets of 20 questions, each with its own psychometric evaluation. Given the objectives of the analysis, and consistent with prior research [10], only the trait-anxiety subscale (T-STAI) was used. Trait anxiety measured by T-STAI was reported to be highly correlated with generalised anxiety disorder [30]; the French version of STAI has been validated in adults from the general population [31]. T-STAI contains 20 items scored on a 4-point Likert scale ranging from “Almost never” to “Almost always.” The higher the score, the greater the proneness to anxiety. As in prior studies, participants were categorised in the “high trait anxiety” group if their total score was equal to or exceeded 40 points or in the “low trait anxiety” group if their total score was below 40 points [32], given that no universally-established cut-off exists. 

### 2.4. Covariates

A validated socio-demographics and lifestyle [33] questionnaire provided self-report data on age, sex, educational level, socio-professional category, marital status, alcohol consumption, and smoking status (variable categories presented in Table 1). Physical activity during the past seven days was assessed with the International Physical Activity Questionnaire and scoring followed established guidelines [34]. Height and weight were self-reported through a validated anthropometrics questionnaire [35]. As these questionnaires are administered at baseline and annually thereafter, for the covariate data for each participant a 2.5-year window around the T-STAI date of completion was applied. Finally, the number of available 24-h dietary records was also modelled as a covariate.

### 2.5. Statistical Analysis

Body mass index (BMI, kg/m^2^) was calculated based on the self-reported height and weight. Next, for covariables with >5% missing values, a “Missing data/not reported” category was created. Specifically regarding the variable “socio-professional category,” if the value was missing and age was <25 or >60 years, the respective status of “student” and “retired” was attributed. Descriptive characteristics of the high and low trait anxiety groups are presented as percentages from chi-squared tests for categorical variables and as mean (±SD) values from Student *t*-tests for continuous variables. For the main analyses, the dependent variables pertained to mean carbohydrate/sugar intake (modelled on a continuous scale) and the main independent variable was trait anxiety (modelled as a two-category variable representing high and low trait anxiety). The associations of interest were evaluated by comparison tests using analysis of covariance (ANCOVA). Model 1 was adjusted for mean total energy intake (Kcal/d, continuous scale), age (years, continuous scale), and sex. Model 2 was adjusted for mean total energy intake (Kcal/d, continuous scale), age (years, continuous scale), sex, BMI (kg/m^2^, continuous scale), alcohol consumption (g ethanol/d, continuous scale), smoking status (never, former, current smoker), physical activity level (low, moderate, high), socio-professional category (homemaker/disabled/unemployed/student, manual/blue collar/office work/administrative staff, professional/executive staff, retired), marital status (living alone or married/cohabiting), and number of 24-h dietary records (continuous scale). Tests for interaction (significance level *p* < 0.10) by age, sex, BMI, and smoking status were also performed. All tests were two-sided and *p* < 0.05 was considered as evidence for statistical significance in the main analysis. SAS version 9.4 (SAS Institute, Inc., Cary, NC, USA) was used for all analyses.

## 3. Results

### 3.1. Description of the Study Population

**Figure 1:**
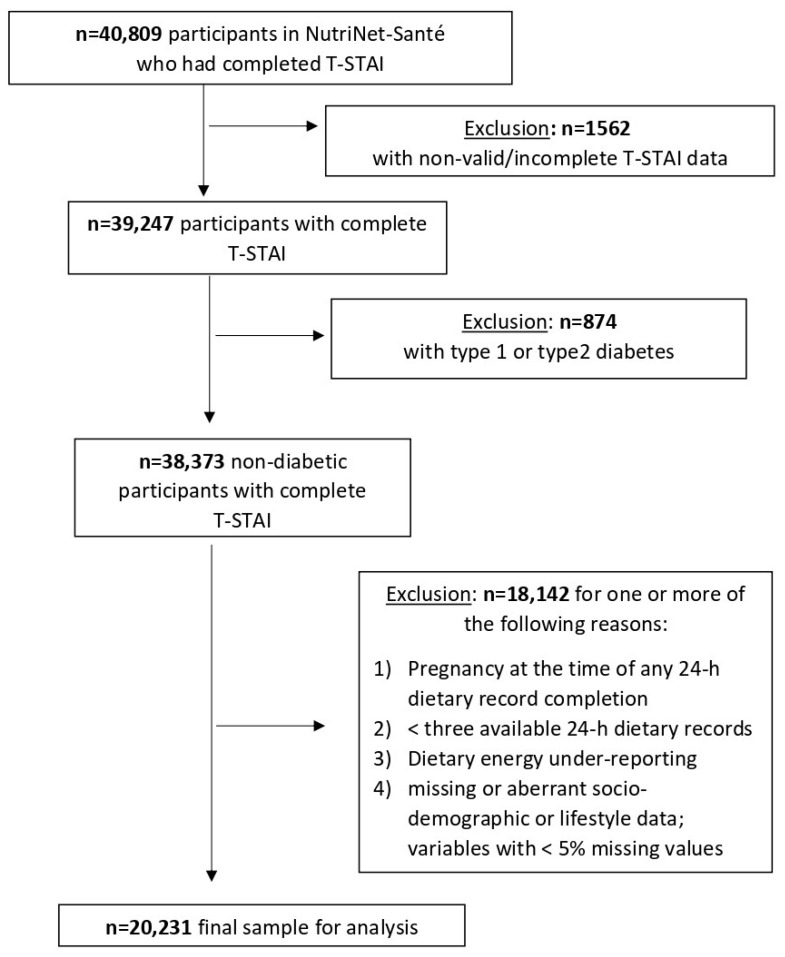
Participant selection flowchart.

In total, 40,809 NutriNet-Santé participants completed the T-STAI. Among them, 1562 participants had some non-valid, missing or partial data and were excluded from the analyses. Among the remaining participants, those with prevalent or incident diabetes type 1 or type 2 were ineligible for the study (n = 874). Next, a total of 18,142 individuals were excluded from the analysis due to one or more of the following reasons: (1) pregnancy at the time of any 24-h dietary record completion, (2) <3 available 24-h dietary records, (3) dietary energy under-reporting, (4) <5% missing or incomplete socio-demographic and/or lifestyle data. Thus, 20231 participants were included in the final sample for analysis (Figure 1). Descriptive characteristics of the high and low trait anxiety groups are presented in Table 1. Overall, 39.3% (n = 7942) of the sample was categorised in the high trait anxiety group. They were more likely to be women, younger, living alone, and underweight compared to those in the low trait anxiety group. Likewise, there were more individuals without any professional activity, with low physical activity, and current smokers in the high trait anxiety group in comparison to the low trait anxiety group. With regard to educational level, no significant difference was observed between the two groups. Mean daily alcohol consumption, total energy, and number of 24-h dietary records of the high trait anxiety group were significantly lower than those of the low trait anxiety group. In the full simple, the mean number of 24-h dietary records was 7.0 ± 2.8.

### 3.2. Comparison of Sugar Intake Across Trait Anxiety Status

Given significant interaction results for age (*p* < 0.08), which is also consistent with evidence in the literature indicating that anxiety tends to decline with age [1,3], the main results are presented by age group (<45 versus ≥45 years). Table 2 (age < 45 years) and Table 3 (age ≥ 45 years) show adjusted comparisons of mean carbohydrate/sugar consumption between high and low trait anxiety groups obtained via ANCOVA. In the younger age group, the partially and fully adjusted analyses produced similar results. In the fully-adjusted analysis (Model 2), individuals aged under 45 years with high trait anxiety had significantly higher mean consumption of added simple sugars compared to those with low trait anxiety (43.9 versus 42.3 g/d; *p* < 0.0007). In turn, in the older age group, two out of four partially-adjusted significant associations were attenuated and became non-significant following full adjustment. Among those aged over 45 years, the results of Model 2 revealed that individuals with high trait anxiety had significantly lower mean consumption of fresh fruit (214.0 versus 219.5 g/d; *p* < 0.02) and lower overall consumption of sweet food/beverages (414.1 versus 420.1 g/d; *p* < 0.03) compared to those with low trait anxiety.

## 4. Discussion

This epidemiological study, conducted in a large sample of French non-diabetic adults, revealed age-specific associations between trait anxiety and sugar intake. Specifically, individuals aged <45 years with high trait anxiety consumed more added simple sugars whereas those aged ≥45 years consumed less fresh fruit and sweet food/beverages in comparison with their low trait anxiety counterparts. No significant differences were observed between the two anxiety groups as regards the percentage of energy from carbohydrates, mean intake of total/complex carbohydrates, simple sugars, or the sweet food/beverage groups excluding fresh fruit. These null findings might be partly attributed to reduced variability in dietary intake owing to potential selection bias. Overall, NutriNet-Santé participants are considered health and nutrition-conscious adults who are more likely to belong to higher socio-economic categories compared with the general population [36]. Next, it has been suggested that fruit intake might have a protective association with anxiety among adults [5,6], which is consistent with our findings, especially among those aged ≥45 years. The bioactive compounds in fruit such as vitamins, fibres, and antioxidants likely have a beneficial impact on mental health in general and on anxiety status in particular. The significant difference as regards the sweet food/beverage group (including fresh fruit) in those aged ≥45 years was mainly driven by consumption of fresh fruit, as no difference was detected in the consumption of the sweet food/beverage group, which excluded fresh fruit.

To our knowledge, no previous study with a large and heterogeneous sample has reported findings on the relationship between the consumption of added simple sugars and anxiety. A cross-sectional study with 1956 college students in one U.S. region had previously reported that added sugars intake was a significant predictor of anxiety in females [21]. Nonetheless, given that ours was a cross-sectional study, the observed association could be bidirectional. As trait anxiety is regarded as a stable disposition and was modelled as the exposure variable, the observed increased intake of added simple sugars (i.e., sucrose, fructose, glucose, high fructose corn syrup or concentrated fruit juices: sugars added during food processing [37]) among relatively young adults with high trait anxiety could be interpreted as compensatory behaviour intended to provide a sense of comfort and well-being. In fact, studies have reported that stress and emotional arousal induced palatable food consumption in humans [17,38]. Our results are consistent with those of a recent German study showing that mental health problems predicted soft drink consumption over six years, but not vice versa [39]. These authors regarded soft drink consumption as a dysfunctional coping strategy [39]. Consumption of palatable food, especially simple sugars, induces dopamine secretion, which is involved in the reward pathway. Thus, intake of simple sugars can enhance the level of perceived pleasure [18]. Interestingly, it was reported that young people exhibited negative affect, including feelings of anxiety, prior to consuming high-calorie sweet foods, whereas comfort food consumption was more likely to be triggered by positive affect in older people [38]. Such results are consistent with the age-specific associations observed in this study. In addition, anxiety disorders typically emerge earlier in life and tend to decrease with age [1,3].

In turn, studies have reported the influence of sugars on inflammation, gut microbiome dysbiosis and brain insulin resistance, which are well established as mechanisms underlying anxiety disorders. For example, prior research has reported a relationship between sugar consumption and low-grade inflammation [15]. There is also evidence that peripheral pro-inflammatory cytokines could generate neuroinflammation that may influence anxiety status by modifying neurotransmitter synthesis, neuroendocrine function, and neurocircuits [16]. In terms of the gut microbiome, a recent review showed that gut microbes were influenced by changes in the consumption of sugars; the authors suggested that alterations in the sugar content in the gut induced transcriptional, compositional, and/or genetic changes in gut microbes [40]. Furthermore, certain gut microbes are directly involved in the production of neurotransmitters such as dopamine and can impact host production of serotonin [41], which could affect anxiety status. 

Given the considerable burden of anxiety disorders [1,2] and their high comorbidity with other mental and physical conditions [3,4], epidemiological and prevention research is indispensable in order to accumulate evidence to guide public health policy. Our study revealed a positive association between the consumption of added simple sugars and trait anxiety, which is highly correlated with anxiety disorders, including generalised anxiety disorder [30]. 

Limitations of this study should be acknowledged. Because of the cross-sectional design, causality could not be tested or inferred. Future prospective research could address not only questions of causality but also the potential bidirectional association between sugar intake and anxiety proneness. Next, we used a literature-based cut-off of 40 points on the T-STAI to identify individuals with high trait anxiety [32], yet the STAI was originally designed as a continuous scale and there is no validated cut-off value. In addition, our assessment of trait anxiety does not correspond to any clinical diagnoses of anxiety disorders, which generally concern a lower percentage of the population than that observed in this study. Indeed, 39.3% of the participants were categorised in the high trait anxiety group in the present study, whereas pooled estimates have suggested that lifetime anxiety disorders affect about 17% of adults [1]. However, the estimate of trait anxiety prevalence in our study was consistent with that of previous research using a T-STAI cut-off of 40 points [32]. Further, despite the statistical adjustment for a large number of pertinent covariables, residual confounding by unmeasured/unavailable constructs (e.g., ethnoracial status, family history, low-sugar dieting) could not be excluded. Finally, in NutriNet-Santé study, women and individuals of high socio-economic status are over-represented as compared with the general population [36], which may partly explain the observed small effect sizes. Whereas the sample for analysis was very large and heterogeneous (a marked strength of the study), only 34% of the solicited NutriNet-Santé participants returned a completed STAI questionnaire; of that sample, a further 49.5% were excluded owing to missing/aberrant data or not meeting the inclusion criteria for this study. Therefore, caution should be used when generalising the findings.

Despite these limitations, the study presents several strengths. To our knowledge, it is the first large epidemiological study to report an association between added simple sugars intake and anxiety status. In addition, data were collected by validated instruments in a large and heterogeneous sample of adults. Dietary sugar intake in particular was calculated on the basis of a mean of seven 24-h dietary records per participant. Furthermore, the dietary intake comparisons between individuals with high versus low trait anxiety were controlled for a number of pertinent covariates. 

## 5. Conclusions

This large cross-sectional study revealed modest age-specific associations between trait anxiety and sugar intake among non-diabetic adults. Specifically, individuals aged under 45 years with a high proneness to anxiety consumed more added simple sugars compared to those with a low proneness to anxiety. In turn, the quantity of fresh fruit intake appeared to be lower among adults aged over 45 years with high versus those with low trait anxiety. Prospective studies with representative samples and possibly even diet-focused randomised controlled trials could elucidate causality and the potential bi-directionality of the observed associations.

## Figures and Tables

**Table 1:** Descriptive characteristics of NutriNet-Santé participants according to trait anxiety status (n = 20,231).

	Full Sample n = 20,231		Low Trait Anxiety (T-STAI < 40) n = 12,289		High Trait Anxiety (T-STAI ≥ 40) n = 7942		*p* Value ^1^
**T-STAI score** ^2^, mean (SD)	37.9	(10.0)	31.4	(5.2)	48.0	(6.9)	<0.0001
**Sex**							<0.0001
Male	5198	(25.7)	3782	(30.8)	1416	(17.8)	
Female	15,033	(74.3)	8507	(69.2)	6526	(82.2)	
**Age, years, mean (SD)**	53.7	(13.6)	55.1	(13.3)	51.6	(13.9)	<0.0001
**Age category**							<0.0001
<35 y	2358	(11.7)	1181	(9.6)	1177	(14.8)	
35–54 y	7109	(35.1)	4036	(32.8)	3073	(38.7)	
55–64 y	5472	(27.0)	3441	(28.0)	2031	(25.6)	
≥65 y	5292	(26.2)	3631	(29.6)	1661	(20.9)	
**Educational level**							0.80
Less than high school	2908	(14.4)	1755	(14.3)	1153	(14.5)	
High school diploma or equivalent	3586	(17.7)	2188	(17.8)	1398	(17.6)	
College, undergraduate degree	5461	(27.0)	3286	(26.7)	2175	(27.4)	
Graduate degree	6988	(34.5)	4271	(34.8)	2717	(34.2)	
Not reported	1288	(6.4)	789	(6.4)	499	(6.3)	
**Socio-professional category**							<0.0001
Homemaker/disabled/	2255	(11.2)	1157	(9.4)	1098	(13.8)	
unemployed/student/trainee	6726	(33.3)	3802	(30.9)	2924	(36.8)	
Manual/blue collar/office	4714	(23.3)	2909	(23.7)	1805	(22.7)	
Professional/executive staff Retired	6536	(32.3)	4421	(36.0)	2115	(26.6)	
**Marital status**							<0.0001
Living alone (single, divorced, widowed)	4827	(23.9)	2716	(22.1)	2116	(26.6)	
Married/cohabiting	15,404	(76.1)	9573	(77.9)	5831	(73.4)	
**Physical activity ^3^**							<0.0001
Low	3588	(17.7)	2001	(16.3)	1587	(20.0)	
Moderate	7541	(37.3)	4549	(37.0)	2992	(37.7)	
High	6589	(17.7)	4354	(35.4)	2236	(28.1)	
Not reported	2513	(12.4)	1385	(11.3)	1128	(14.2)	
**Body Mass Index (BMI,** **kg/m^2^), mean (SD)**	23.6	(3.9)	23.7	(3.7)	23.4	(4.1)	<0.0001
**BMI category**							<0.0001
Underweight (<18.5)	917	(4.5)	455	(3.7)	462	(5.8)	
Normal weight (18.5–24.9)	13,401	(66.2)	8171	(66.5)	5230	(65.9)	
Overweight (25.0–29.9)	4693	(23.2)	2945	(24.0)	1748	(22.0)	
Obese (≥30)	1220	(6.0)	718	(5.8)	502	(6.3)	
**Smoking status**							<0.0001
Never smoker	10,234	(50.6)	6098	(49.6)	4136	(52.1)	
Former smoker	7867	(38.9)	4993	(40.6)	2874	(36.2)	
Current smoker	2130	(10.5)	1198	(9.8)	932	(11.7)	
**Alcohol use,** **g ethanol/d, mean (SD)**	8.5	(11.5)	9.1	(11.9)	7.5	(10.8)	<0.0001
**Total energy intake,** **Kcal/d, mean (SD)**	1910.5	(441.5)	1933.5	(449.0)	1874.9	(427.1)	<0.0001
**Number of 24-h dietary record, mean (SD)**	7.0	(2.8)	7.1	(2.8)	6.8	(2.8)	<0.0001

Values refer to number (%) except when noted otherwise. ^1^ *p*-values obtained from chi-squared tests or Student *t*-tests, as appropriate. ^2^ Spielberger Trait Anxiety Inventory (T-STAI), form Y; score range between 20 and 80 points, with higher scores reflecting higher proneness to anxiety. ^3^ Assessed with the International Physical Activity Questionnaire-Short Form according to established scoring criteria.

**Table 2:** Comparison of mean daily intake of sugar between high and low trait anxiety groups (non-diabetic adults aged <45 years; n = 5500).

	Model 1 ^1^					Model 2 ^2^				
	T-STAI ^3^ < 40		T-STAI ^3^ ≥ 40		*p* Value	T-STAI ^3^ < 40		T-STAI ^3^ ≥ 40		*p* Value
	n = 2902		n = 2598			n = 2902		n = 2598		
	LSmean	SE	LSmean	SE		LSmean	SE	LSmean	SE	
Percentage energy from carbohydrates	42.05	0.11	42.19	0.12	0.40	42.57	0.81	42.71	0.81	0.36
Total carbohydrates (g/d)	198.86	0.53	199.30	0.56	0.58	201.71	3.78	202.16	3.80	0.53
Complex carbohydrates (g/d)	104.54	0.43	104.49	0.45	0.94	105.53	3.28	105.54	3.31	0.97
Simple sugars (g/d)	93.73	0.43	94.21	0.45	0.50	95.60	3.15	96.03	3.17	0.46
Added simple sugars (g/d)	41.40	0.33	43.15	0.35	0.0003	42.33	2.50	43.92	2.51	0.0007
Sweet food/beverage group (g/d)	429.08	3.02	427.08	3.19	0.65	398.28	22.85	396.31	22.97	0.64
Sweet food/beverage group except fresh fruit (g/d)	272.69	2.52	274.40	2.67	0.46	248.36	19.45	250.43	19.55	0.57
Fresh fruit (g/d)	157.39	2.36	152.67	2.50	0.17	149.92	18.14	145.88	18.24	0.23

^1^ ANCOVA adjusted for mean total energy intake, age, and sex. ^2^ ANCOVA adjusted for mean total energy intake, age, sex, BMI, alcohol consumption, smoking status, physical activity level, socio-professional category, marital status, and number of 24-h dietary records. ^3^ Spielberger Trait Anxiety Inventory (T-STAI), form Y; score range between 20 and 80 points, with higher scores reflecting higher proneness to anxiety.

**Table 3:** Comparison of mean daily intake of sugar between high and low trait anxiety groups (non-diabetic adults aged ≥45 years; n = 14,731).

	Model 1 ^1^					Model 2 ^2^				
	T-STAI ^3^ < 40		T-STAI ^3^ ≥ 40		*p* Value	T-STAI ^3^ < 40		T-STAI ^3^ ≥ 40		*p* Value
	n = 9387		n = 5344			n = 9387		n = 5344		
	LSmean	SE	LSmean	SE		LSmean	SE	LSmean	SE	
Percentage energy from carbohydrates	40.88	0.07	41.06	0.09	0.11	41.09	0.09	41.23	0.11	0.15
Total carbohydrates (g/d)	195.83	0.33	196.40	0.44	0.30	196.73	0.45	197.04	0.51	0.51
Complex carbohydrates (g/d)	102.32	0.26	103.22	0.34	0.04	104.23	0.40	104.91	0.45	0.10
Simple sugars (g/d)	92.92	0.25	92.59	0.33	0.43	91.93	0.37	91.55	0.42	0.32
Added simple sugars (g/d)	35.02	0.17	35.61	0.23	0.04	35.12	0.27	35.56	0.31	0.12
Sweet food/beverage group (g/d)	427.80	1.69	421.55	2.25	0.03	420.08	2.59	414.06	2.94	0.03
Sweet food/beverage group except fresh fruit (g/d)	200.32	1.14	200.30	1.52	0.99	200.53	1.81	200.10	2.05	0.82
Fresh fruit (g/d)	227.48	1.46	221.25	1.95	0.01	219.54	2.29	213.97	2.59	0.02

^1^ ANCOVA adjusted for mean total energy intake, age, and sex. ^2^ ANCOVA adjusted for mean total energy intake, age, sex, BMI, alcohol consumption, smoking status, physical activity level, socio-professional category, marital status, and number of 24-h dietary records. ^3^ Spielberger Trait Anxiety Inventory (T-STAI), form Y; score range between 20 and 80 points, with higher scores reflecting higher proneness to anxiety.

## Data Availability

Data used in this study are under the protection of national health data regulations set forth by the French National Commission on Informatics and Liberty (Commission Nationale de l’Informatique et des Libertés, CNIL) which prohibit free public access. The data can be made available upon written request sent to the NutriNet-Santé operational coordinator, Nathalie Druesne-Pecollo (n.pecollo@eren.smbh.univ-paris13.fr), and following approval by the NutriNet-Santé steering committee.
